# Assessment of Variability Sources in Grape Ripening Parameters by Using FTIR and Multivariate Modelling

**DOI:** 10.3390/foods12050962

**Published:** 2023-02-24

**Authors:** Daniel Schorn-García, Barbara Giussani, María Jesús García-Casas, Daniel Rico, Ana Belén Martin-Diana, Laura Aceña, Olga Busto, Ricard Boqué, Montserrat Mestres

**Affiliations:** 1 Instrumental Sensometry (iSens), Department of Analytical Chemistry and Organic Chemistry, Campus Sescelades, Universitat Rovira i Virgili, Edifici N4, C/Marcel⋅lí Domingo s/n, 43007 Tarragona, Spain; 2 Dipartimento di Scienza e Alta Tecnologia, Università Degli Studi Dell’Insubria, Via Valleggio, 9, 22100 Como, Italy; 3 Consejería de Agricultura y Ganadería, Finca de Zamadueñas, Ctra. Burgos km. 119, 47171 Valladolid, Spain; 4 Chemometrics, Qualimetrics and Nanosensors Group, Department of Analytical Chemistry and Organic Chemistry, Campus Sescelades, Universitat Rovira i Virgili, Edifici N4, C/Marcel⋅lí Domingo s/n, 43007 Tarragona, Spain

**Keywords:** portable MIR, variability, ASCA, process monitoring, precision viticulture

## Abstract

The variability in grape ripening is associated with the fact that each grape berry undergoes its own biochemical processes. Traditional viticulture manages this by averaging the physicochemical values of hundreds of grapes to make decisions. However, to obtain accurate results it is necessary to evaluate the different sources of variability, so exhaustive sampling is essential. In this article, the factors “grape maturity over time” and “position of the grape” (both in the grapevine and in the bunch/cluster) were considered and studied by analyzing the grapes with a portable ATR-FTIR instrument and evaluating the spectra obtained with ANOVA–simultaneous component analysis (ASCA). Ripeness over time was the main factor affecting the characteristics of the grapes. Position in the vine and in the bunch (in that order) were also significantly important, and their effect on the grapes evolves over time. In addition, it was also possible to predict basic oenological parameters (TSS and pH with errors of 0.3 °Brix and 0.7, respectively). Finally, a quality control chart was built based on the spectra obtained in the optimal state of ripening, which could be used to decide which grapes are suitable for harvest.

## 1. Introduction

Wine quality is strongly dependent on the characteristics of the grapes used to elaborate it; therefore, to guarantee their optimal state, a quality control is performed regularly during their ripening process [1]. The common oenological parameters used to follow the evolution of ripening are sugar content and pH, although more detailed information about the ripeness and quality of the grapes can be obtained by determining their phenolic composition, total acidity, texture and flavor [2]. Setting the optimal ripening point is a key factor in achieving maximum quality in any fruit, but even more so when it comes to non-climacteric fruits such as grapes. This implies that if samples are collected too early, no improvement in any of their quality parameters will be achieved [3], and conversely, if grapes are harvested too late, postharvest disorders are more likely to occur [4].

Another important aspect to consider when working with grapes is the lack of uniformity in the evolution of oenological parameters within the vineyard. This fact has already been shown by different studies in which it has been confirmed that the accumulation of sugars [3,5], anthocyanins [6] or phenolic compounds [7] is not regular in the grapes of the same vineyard [8]. These differences are mainly due to the different viticultural practices—such as soil preparation and tilling, trellising, and pruning of vines or treatments to fight diseases—and to climate and exposure to sunlight, which produces different physiological responses in each plant, in each bunch/cluster and even in each grape [9]. This individual behavior of the grape has led traditional viticulture to so-called precision viticulture, which seeks to know the characteristics of each plant within the vineyard and its evolution over time [10]. However, to obtain this detailed knowledge of the vineyard, it is necessary to carry out a very rigorous and exhaustive sampling followed by a rapid and reliable analysis of the oenological parameters. This entails high costs, both in time and money, so the new trends are focused towards the use of portable (to be able to use them in situ in the field) and rapid analysis techniques together with appropriate statistical treatments that allow choosing those variables that provide significant information to follow the process of interest.

Among the analytical techniques that can be applied on site, vibrational spectroscopy is gaining acceptance because it is fast, robust, and portable [11]. Moreover, as the spectra obtained contain information from almost-all chemical bonds in the sample, when coupled with chemometric data analysis, it provides qualitative and quantitative information on the composition of the samples under study, often through classification and prediction models [12]. Regarding grape berries, vibrational spectroscopy proved to be suitable for the prediction of several parameters related to technological and phenolic maturities (see [13] and references therein). Additionally, there are chemometric tools that decompose and evaluate the effects of known factors in an experimental design over the multivariate instrumental response such as ANOVA–simultaneous component analysis (ASCA) [14]. ASCA has been applied to spectroscopic data to study the sources of variability during coffee roasting under different conditions [15] or to evaluate chilling injury on the aubergine fruit [16]. Other authors have used ASCA as an exploratory tool to understand which analytical factors directly affect the measurement. Thus, Amigo et al. have shown the influence of the measurement area on near-infrared spectra of bread [17] and Borraz-Martínez et al. studied the influence of sampling on the discrimination of different varieties of *Prunus dulcis* leaves [18].

The aim of the present work is threefold. First, assessing the variability sources that affect grape ripeness in terms of harvest time and relative position (both on the plant and on the bunch/cluster) using Fourier transform infrared (FTIR) portable spectroscopy and multivariate analysis. ASCA was used to evaluate the contribution of the different factors and to study their evolution along the maturity process. Secondly, building prediction models of the main parameters related to technological maturity (TSS and pH) from the spectra of the grapes recorded during ripening. Thirdly, proposing a strategy based on multivariate statistical process control to determine which grapes have the optimal quality at each moment.

## 2. Material and Methods 

### 2.1. Vineyard and Maturity Control

The samples used in this study were obtained from the experimental vineyard of the Faculty of Oenology (Universitat Rovira i Virgili, Spain) located in the Mas dels Frares center (Constantí, Spain) (41°08′44′′ N 1°12′02′′ E; Altitude: 60 m; 15 km from the Mediterranean Sea). The climate is characterized by high ambient humidity (60–70%) with hot, dry summers and mild, wet winters. Since the experimental cellar has a record of the evolution of each variety, which includes the harvest date of each vintage, maturity controls usually begin three weeks before the average harvest date. To guarantee the optimal condition of the grapes, these controls involve the measurement of both technological and phenolic maturity. Technological maturity is determined by measuring the pH and the potential alcoholic strength by the volume (ABV) of the grapes [1], while phenolic maturity is determined by measuring the polyphenol content with the Slinkard and Singleton method [19] and the sensory quality by evaluating the hardness of the skin and the color of the seeds [1]. In addition, taking into account the relevance of the choice of the optimal harvest day for this study, it was decided that the quality of the polyphenols would be evaluated by measuring their antioxidant capacity. Specifically, the oxygen radical absorbance capacity (ORAC) following the method described by Jiménez-Pulido et al. [20] was determined.

### 2.2. Samples and Sampling

Ninety grape berries of the variety ‘*Muscat of Alexandria*’ were harvested during the entire ripening process. The vineyards are 9 years old and cover 0.65 ha of the Mas dels Frares experimental vineyard, which has a climate that offers the optimal conditions to achieve adequate levels of sugar in the berries of this variety, which are larger than average. In this way, it was guaranteed that the ripening process would develop correctly and that the samples collected would allow obtaining enough juice to carry out the analyses of each berry separately. 

A comprehensive sampling was performed to ensure the full monitoring of the ripening process (including overripe samples). Sampling was carried out by collecting 18 grape berries at five different times from 12 August to 15 September (about once a week), as the harvest date was 7 September. This date was considered optimal because the parameters of ripeness, both technological and phenolic, remained practically constant when compared with the values found the previous week and because, although not significant, even a negative trend was sensed in the phenolic parameters (Table 1).

Grape berries were identified according to the sampling date, the position of the bunch on the plant (top, center, and bottom) and according to the position of the berry within the bunch (top, center, and bottom). Grapes were collected from different plants and bunches to obtain a representative sample of the vineyard. In all cases, a duplicate of the same position relative to plant and bunch, but on different plants, was considered as a biological replicate to ensure maximum variability.

The samples were kept cold until the analysis in portable refrigerators, making sure that the pedicel remained intact to prevent any degradation or evolution.

### 2.3. Determination of Total Soluble Solids and pH in Individual Berries

Once harvested, the samples were immediately taken to the lab, where each individual grape berry was gently crushed with a small manual garlic crusher and the juice obtained was poured directly into 2 mL plastic containers. Analyses of sugar content, expressed as total soluble solids (TSS) were performed at room temperature by using an automatic temperature compensation digital handheld refractometer (HI 96801, Hanna instruments). Before use, the refractometer was calibrated with deionized water and the crystal was thoroughly cleaned with deionized water and wiped dry with cellulose tissues before each new reading. The pH was measured directly into the plastic container with a portable pH meter with a Micro P portable electrode (7 + series portable pH-meter, XS Instruments, Italy). Before analysis, the pH meter was calibrated with two reference standards (pH 7.00 and 4.00).

### 2.4. Mid-Infrared Spectroscopic Analysis

MIR analyses were carried out using a portable 4100 ExoScan FTIR instrument (Agilent, CA, USA), equipped with an interchangeable spherical attenuated total reflectance (ATR) sampling interface consisting of a diamond crystal window and with a diffuse reflectance sampling interface (DRIFT). All samples were analyzed with both interfaces, since the ATR was used to measure the crushed grapes and the DRIFT was used to measure the entire grape berry before crushing.

The spectra were acquired with the Microlab PC software (Agilent, CA, USA) using a methodology previously optimized [21], that is, measuring from 4000 to 650 cm^−1^, with 32 scans and 8 cm^−1^ resolution.

When using the ATR sampling interface, a drop of each sample (without any pre-treatment) was placed onto the crystal using a plastic Pasteur pipette, ensuring the complete coverage of the ATR crystal, and the spectrum was acquired right afterwards. Each sample was analyzed in triplicate, using three different drops of the crushed grape berry. After each measurement, the crystal was carefully cleaned using deionized water and dried with cellulose tissues. To ensure reliable results, an air background was carried out before each triplicate, i.e., one background before each sample.

For berry measurements using the DRIFT interface, the spectrometer was placed vertically and the grapes were supported in the sampling gap. Each sample was analyzed in triplicate by placing the grape berry in three different positions (two in the horizontal plane and one opposite the pedicel). In this case, the background was acquired with a 100 Micron Reference (Agilent, CA, USA) after every triplicate.

### 2.5. Data Analysis

#### 2.5.1. Spectral Data Pre-Processing

Spectra were imported with MATLAB (R2021a, 9.10; MathWorks, Natick, MA, USA) to create two datasets (ATR-FTIR and DRIFT), each consisting of 270 spectra (triplicates of ninety samples) and 845 wavelengths. All multivariate analyses were performed using the PLS Toolbox v9.0 (Eigenvector Research Inc., Wenatchee, WA, USA). Different pre-processing combinations were tested to mitigate the noise and baseline drifts observed in the raw spectra. The combination that provided the best results for the different models was second order polynomial Savitzky–Golay (SG) smoothing and standard normal variate (SNV). SG is useful for reducing spectral noise, but special attention must be paid to the window size, as severe smoothing could remove useful information from the spectra. SNV was applied to correct spectral light scattering caused by the physical aspects of the sample or equipment characteristics [22,23]. Finally, after spectral pre-processing, data were mean-centered.

#### 2.5.2. Principal Component Analysis (PCA)

PCA was first used to visualize the data and identify outliers by interpreting the score plot and the Hotelling T^2^ vs. Q residuals plot (Figure S1). PCA is an exploratory tool that reduces the dimensionality of the data while keeping the maximum information. This algorithm decomposes the data into a new set of latent variables called principal components, which are linear combinations of the original variables and retain the most information. The projections of the samples onto the new space of principal components (PCs) are called scores, and the angles/projections between the original variables and the PCs are called loadings. Exploring the score and loading plots provides a better understanding of the sources of variability in the spectra and can reveal clusters and trends in the data.

#### 2.5.3. Partial Least Squares (PLS) Regression

PLS regression was applied to predict TSS and pH in the collected samples. The average of the three replicate spectra was used to build the PLS models. An X matrix of spectra (90 samples × 845 wavelengths) was correlated to two y vectors (90 × 1) containing the values for TSS and pH, respectively. For TSS prediction, two approaches were attempted: (1) Using the whole spectrum; and (2) Selecting specific regions based on a previous study [24], which were 967–1175 cm^−1^ and 1483 to 1771 cm^−1^. To assess model robustness, an external validation was performed. Samples were thus split into calibration and validation sets using the Kennard–Stone [25] and onion [26] algorithms. Two sample splits were used for calibration and validation; half of the data in each set in the first one and 2/3 for calibration and 1/3 for validation in the second one.

The statistical measure root mean square error of prediction (RMSEP) was used (Equation (1)) to evaluate how well the model predicts new samples (not used when building the model):

(1) $$\mathrm{RMSEP}=\sqrt{\frac{\sum_{i}^{n_{t}}{\left(y_{t,i}-{\;\hat{y}}_{t,i}\right)}^{2}}{n_{t}}}$$

${\hat{y}}_{t,i}$ is the pH or °Brix predicted by the model, $y_{t,i}$ is the measured value (actual pH or °Brix), and n_t_ is the number of samples in the test set. RMSEP expresses an average error to be expected in future predictions when the calibration model is applied to unknown samples.

The statistical parameters ratio of performance to deviation (RPD) and range error ratio (RER) were used (Equations (2) and (3)) to evaluate the predictive ability of the models [27]:

(2) $$\mathrm{RPD}=\frac{\mathrm{SD}}{\mathrm{RMSEP}}$$

(3) $$\mathrm{RER}=\frac{y_{\max}-y_{\min}}{\mathrm{RMSEP}}$$

where SD is the standard deviation of the reference parameters, and y_max_ and y_min_ are the maximum and minimum values of the reference parameters. Both RPD and RER are commonly used to describe whether the obtained prediction models are good enough, with RPD > 2 and RER > 10 indicating good predictive ability.

#### 2.5.4. ANOVA–Simultaneous Component Analysis (ASCA)

Analysis of variance (ANOVA)–simultaneous component analysis (ASCA) is a multivariate exploratory method based on the univariate ANOVA, which decomposes the variability sources affecting the data. ASCA decomposes the overall variation into the main effects and their binary combinations, which are included in a matrix according to a predefined experimental design [28]. The variability sources considered in this study were: (1) Sampling date, which is directly related to the ripening time or maturity status (5 levels); (2) Position of the bunch in the plant (3 levels); (3) Position of the grape within the bunch (3 levels); (4) Spectroscopic replicates (3 levels) and their interactions. The first step of ASCA involves partitioning the centered matrix **X**_c_ according to Equation (3):

(4) $$X_{c}=X-1m^{T}=X_{\mathrm{Ripening}}+X_{\mathrm{pos}.\mathrm{plant}}+X_{\mathrm{pos}.\mathrm{bunch}}+X_{\mathrm{Ripening}\;x\;\mathrm{pos}.\mathrm{plant}}+X_{\mathrm{Ripening}\;x\;\mathrm{pos}.\mathrm{bunch}}+X_{\mathrm{pos}.\mathrm{plant}\;x\;\mathrm{pos}.\mathrm{bunch}}+X_{\mathrm{res}}$$

where **1** is a vector of ones, **m**^T^ is the average spectrum of the samples, **X**_Ripening_, **X**_pos.plant_, **X**_pos.bunch_ are the matrices of the main factors, **X**_Ripening x pos.plant_, **X**_Ripening x pos.bunch_, **X**_pos.plant x pos.bunch_ are the effect matrices for the binary interactions, and **X**_res_ is the residual matrix collecting all the variability not accounted in the experimental design. Each matrix is centered and contains the mean profiles of the samples corresponding to each factor or interaction level. As an example, if the ripening factor has five levels with 18 observations each, the 18 observations will contain the average profile for the first level of the ripening factor and the same will happen with next level, and so on. The interaction matrix is calculated after the subtraction of the main effect matrices. Afterwards, each matrix is decomposed using simultaneous component analysis (SCA), which reduces to standard principal component analysis under the constrains of ANOVA [14].

#### 2.5.5. Multivariate Statistical Process Control (MSPC) Charts

A PCA-based multivariate statistical process control (MSPC) was applied using the grape spectra of the 4th harvest time, which show the optimal maturity characteristics. Q residuals and Hotelling T^2^ values were calculated and plotted in a control chart with 95% confidence limits. The rest of the harvest times were projected onto the PCA (T^2^ vs. Q plot) model. The Q statistic indicates how well each sample fits the model and the Hotelling T^2^ statistic represents the distance of a given sample to the center of the model [29,30].

## 3. Results and Discussion

### 3.1. Optimization of the Analytical Strategy

Different measurement strategies were tried to obtain suitable instrumental signals. For intact grapes, ATR and DRIFT sampling interfaces were tested (illustrated in Figure S2).

Concerning entire grapes, a problem occurred with the analysis of the most mature samples using ATR. The pressure applied to the sample (needed for a complete contact between the sample and the ATR crystal) caused the skin to break in many experiments. Therefore, we decided not to consider this methodology.

**Figure 1:**
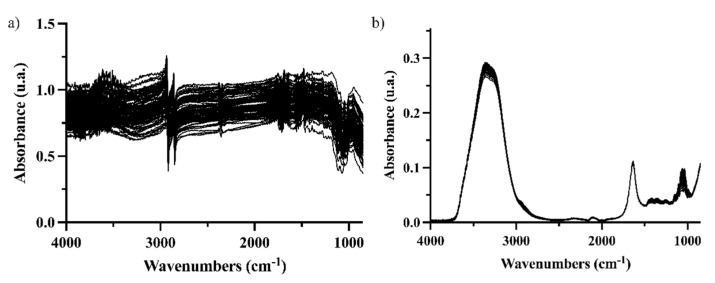
Spectra of 90 grapes using different IR analysis configurations (**a**) DRIFT for intact berries; (**b**) ATR-FTIR for crushed berries.

FTIR coupled to the DRIFT interface was tested on entire grape berries even though a low penetration of the radiation in the sample was expected. It is worth recalling that ripeness implies a softening of the grape skin [5], and this information could be captured by DRIFT-IR spectroscopy. However, spectra were noisy (Figure 1a), especially between 2500 and 4000 cm^−1^.

Individually crushed berries were analyzed with the ATR sampling interface (illustrated in Figure S2), showing great reproducibility for the instrumental replicates and a good signal-to-noise ratio (Figure 1b). For this reason, the final models were built from the ATR-MIR spectra of crushed grapes.

### 3.2. pH and Total Soluble Solids Prediction

The prediction of TSS and pH through multivariate regression required slightly different spectral pre-processing. For the TSS prediction, the pre-processing consisted of SG smoothing with a second-order polynomial and a window width of seven points, followed by SNV. For the pH prediction, the pre-processing consisted of SG smoothing with a second-order polynomial and a window width of fifteen points, followed by SNV. Different validation strategies were applied to assess the robustness of the models [31]. Thus, an external validation set was generated using the Kennard–Stone and onion algorithms, and using different ratios between the calibration and validation sets. The PLS model results are shown in Table 2. No outliers were detected.

As shown by the RMSEP and R^2^ values, when considering the whole spectra range, TSS was successfully predicted in every model regardless of the methodology to select the samples and the ratio of calibration to validation set sizes. It can be stated that just half of the samples analyzed is enough to obtain good prediction results. Regarding the RMSEP, an error of 0.3 °Brix is quite satisfactory since the TSS range considered goes from 10.1 to 25.7 °Brix, and also because after the conversion to potential alcoholic strength, it would mean only an error of ~0.2° (conversion from TSS to ABV according to [32]. A similar TSS error value has been reported (SECV of 0.20 °Brix) in white grape juices [33]. Additionally, the RPD and RER for TSS prediction were 8.1 and 42.7, respectively. Therefore, the prediction ability of TSS is suitable for assessing grape maturity and is consistent with previous results using ATR-MIR in grapes [34].

TSS is strongly related to sugar concentration, so the selection of a specific spectroscopic range related to this parameter was likely to produce better results [24]. The results obtained did not show statistically better predictions in terms of RMSEP, R^2^, RPD or RER, and even poorer models were obtained when the onion sample selection algorithm was used. However, as for the number of factors, the models built with the selected variables needed two factors instead of three. This could be expected as less spectral information is used for prediction.

In the case of pH prediction, the whole spectra range were used, as previous research showed that it provided the best models [24]. This is because variations in pH produce changes in the chemical matrix of the sample, due to changes in bond conformation and matrix properties. As can be seen in Table 2, the prediction error obtained was between 0.06 and 0.07, which is very satisfactory considering the range of the pH values of the samples (between 2.90 and 3.60). This result is comparable to the standard error of cross-validation obtained by Shah et al. to predict pH in grape juice samples [33]. Finally, the best models have an RER of 11.5 and an RPD of 2.34, indicating good prediction ability.

**Figure 2:**
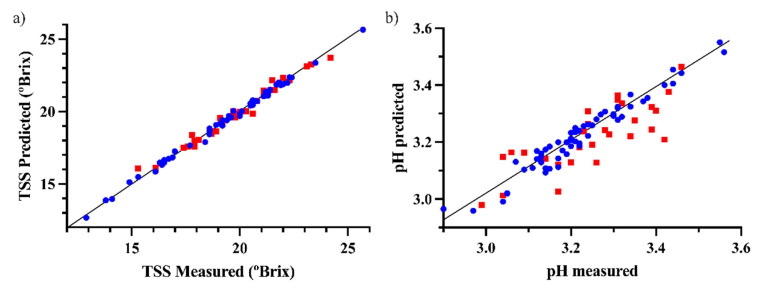
Predicted vs. measured values for the best prediction models of (**a**) TSS and (**b**) pH (blue circles for calibration samples, red squares for validation samples).

The plots of the predicted vs. measured values of the onion external validation using a third of the samples for validation for TSS and pH are depicted in Figure 2.

### 3.3. ANOVA–Simultaneous Component Analysis (ASCA)

An ASCA model was calculated to study the variability sources affecting grapes, using a matrix of 270 samples (90 berries × 3 replicates) and 845 wavelengths (no outliers were detected in the preliminary PCA models).

In the results of an ASCA model, the contribution of each factor in the matrix variability is expressed as a percentage and indicated as % effect. For a given factor, the higher the % effect the more important its contribution. In addition, a permutation test of 10,000 iterations was performed to identify significant factors. The significance of the factor is defined by a *p*-value (a *p*-value under 0.05 means the factor is significant). The ASCA results are summarized in Table 3. 

First, it should be noted that neither the instrumental replica nor its combination with other factors shows a significant effect, demonstrating that the ATR-FTIR portable device has a high reproducibility in these kinds of measurements. All other factors, maturity (sampling date), position in the plant, position in the bunch, and the interactions between these factors, were statistically significant. Nearly a third of the total variance (28.98%) can be attributed to the sampling time effect. This is consistent with the fact that the evolution of the grape along the ripening process comprises a period of 33 days, in which many physical and chemical changes occur in the samples, including the accumulation of free sugars, cations, amino acids, and phenolic compounds [1].

**Figure 3:**
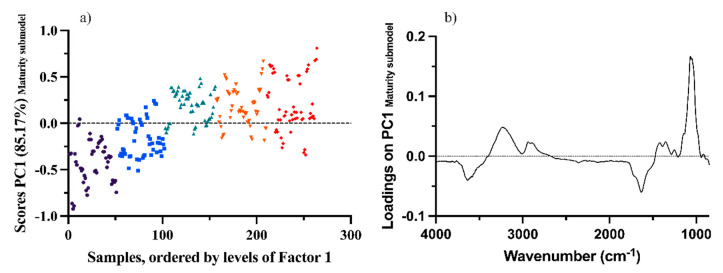
Score (**a**) and loading (**b**) plots of the first factor of the maturity factor submodel. Different colors mean that samples belong to different times (Black—Time 1; Blue—Time 2; Green—Time 3; Orange—Time 4; Red—Time 5).

Figure 3a shows the score values of the maturity submodel for each sample, colored according to different harvest times. Score values are grouped by their sampling time and show an evolution over time. By looking at the first loading of the maturity factor (Figure 3b), this evolution can be assigned to sugars (glucose and fructose), showing a maximum peak at around 1063 cm^−1^ [12,34]. Sugar accumulation and distribution in grapes are major changes in grape samples during ripening [1].

As can be seen in Table 3, the position of the bunch on the plant and the interaction of this factor with the sampling time are the next significant effects in grape spectra after the effect of the sampling time, with an effect of 5.83 and 9.52%, respectively. This result may be due to the different exposure to sunlight depending on the position occupied by the bunch, since there are studies that state that sugar accumulation and final sugar concentration are statistically different depending on the light received by the [35]. Additionally, as the leaves are responsible for photosynthesis, which produces the sugars that are accumulated in grapes, the movement of sugars along the plant can affect the different accumulation of sugars and other metabolites depending on the height of the bunch in the vine [36].

In addition, many authors have stated that grape position in the bunch is a factor of variability in terms of sugar concentration and grape size [37,38]. Pagay and Cheng even found different sugar concentrations in three parts of a cluster/bunch. Their results show that the lower part of the bunch has a significantly different sugar concentration compared to the upper part. ASCA results on Table 3 also show that there is a difference between the different parts of the bunch (2.21% effect), and that this difference evolves over time (5.84% for the interaction between grape position in the bunch and sampling time). ASCA results for the interaction between grape position in the bunch and sampling time are in line with those reported in the study by González-Caballero et al., who investigated the impact of ripeness, position of grapes within the bunch and the orientation of the bunch on near-infrared (NIR) spectra [39]. The study revealed differences in the NIR spectra as a function of ripeness, but also due to the position of the grape within the bunch and the orientation of the bunch.

The first loading of all significant factors shows a large signal from 1187 to 937 cm^−1^, showing that grape ripeness, grape position in the plant, grape position in the bunch, and their interactions are strongly related to the sugar pathway. However, there are other compounds, such as acids, that can also be related to these factors, as there are spectroscopic regions, such as the water band around 3400 cm^−1^ or the fingerprint region between 1800 and 900 cm^−1^, that also contribute to the models (Figure S3a,b).

The variance not explained by the ASCA model (residual term) was of 45.01%. This high value could be explained by many abiotic and biotic factors (pluviometry, temperature, plant side, soil, microorganisms, insect action, etc.), as grapes came from an outdoor field [40].

### 3.4. Sub-ASCA (ANOVA–Simultaneous Component Analysis) Models

As explained above, we found that the influence of the factors changed over time, so five individual ASCA models were built to understand how the relative position of the grapes, both in the plant and in the bunch, evolved with time (Table 3). Five subsets of the original matrix, each consisting of 54 spectra and 845 wavenumbers, were used in each ASCA model (results in Table 4). The effect of the instrumental replicates was confirmed not to be significant in any of the models, nor was the interaction of the instrumental replicates with other factors.

As shown in Table 4, the factors related to grape position evolve over time in a complex manner. The dynamics affecting grape physiology make it difficult to unravel these factors. Grapes, like most fruits, have limited photosynthetic activity, which means that the accumulation of sugars in the berries depends mainly on import from other parts of the plant via the phloem [36]. However, this metabolic input is driven by the individual biochemistry of the grape, as each berry coordinates all the processes necessary in ripening, i.e., sugar accumulation, berry softening, anthocyanin synthesis, metabolism of acids, and accumulation of volatile compounds [5,41]. Therefore, since each berry is responsible for its own ripening process, there is no synchronization between the berries of the grape, even if they are within the same bunch, which leads to additional variability in the system [42].

The results in Table 4 suggest that there is a distribution between the individual factors (position in the plant and position in the bunch) and the interaction between them. Specifically, when the individual factors are larger, the interaction is lower and vice versa. Furthermore, the effect of the position in the plant is the main effect for three of the sampling times considered (2, 4, and 5), and for the other two sampling times (1 and 3) it is the interaction between the position in the plant and the position in the bunch. This may support the idea that the position in the plant is the most significant effect after sampling time, as seen in Table 3. In addition, temperature differences within the grapes, caused by direct and indirect sun exposure and differences in the top and bottom half of a bunch, significantly affect total soluble solids (TSS) and pH levels [43]. These daily temperature fluctuations, which change throughout ripening, contribute to the overall complexity of the evolution of effects over sampling time, as shown in Table 4 [44].

Finally, the residual values indicate that the relative position is the most important factor in the early stages of the ripening process. As time progresses, other factors come into play that cause the residual to increase. This finding suggests that factors other than grape position, such as abiotic and biotic factors, play a more significant role in determining grape physiology as the ripening process progresses.

### 3.5. ANOVA–Simultaneous Component Analysis (ASCA) Model with Reference Parameters

An ASCA model was calculated to study the variability sources affecting grapes in terms of reference parameters. A data matrix of 90 samples and two reference values, pH and TSS, was used for this purpose. Data were autoscaled before applying ASCA. The ASCA results are summarized in Table 5.

ASCA results for pH and TSS show a similar trend to the ASCA results for the spectra, maturity being the most important factor. As shown in Section 3.2, the spectra contain the information of sugars and pH, and this explains the similarity of the results obtained. The effect “position in the plant” could be explained as the difference between the parts of the vine in the metabolites (sugars and acids) imported into the grapes through the phloem [36]. Studies such the one carried out by Doumouya et al. have revealed differences between various parts of the bunch in terms of TSS, pH, TPC, and grape size [45]. The authors emphasize the importance of considering a representative sampling of different parts of the bunch in order to accurately estimate grape ripeness and obtain representative values of the physicochemical properties of the grapes. The small differences between the ASCA models (Table 3 and Table 5) could be explained by the fact that the spectra reflect all molecules in the sample, which are not included in the pH and TSS values, such as anthocyanincs, which have been shown to be a variability source in white grapes [46]. Despite maturity and position in the plant being the only significant factors in the ASCA model for pH and TSS, every factor shows a % effect similar to that of the ASCA built with the spectra (Table 3, see also Table S1 for the results of TSS and pH for each level in each factor). For that reason, the ASCA model obtained with reference parameters confirms the results obtained with the ASCA model for spectra.

### 3.6. Process Control Charts for Ripening Monitoring

As stated in Section 3.3 and Section 3.4, the results of the ASCA models showed that the spatial (position) factors are significant and affect the spectroscopic results. These results have been corroborated using an MSPC approach, based on a control chart built with Q and T^2^ statistics [47].

First, a PCA model was built using the mean spectra (three replicates) of the crushed grape samples belonging to Time 4 (matrix of dimensions 18 × 845). Following the assessment of the viticulturist and based on oenological parameters such as ABV, pH, polyphenolic content and antioxidant activity, Time 4 was selected as optimal for the harvest both in terms of technological and polyphenolic maturity. Two PCs were selected that accounted for around 95% of the variability of the data. The score values of the model were used to calculate the Hotelling T^2^ 95% confidence limit. To calculate the Q residuals, the residual matrix of the PCA model was used. Individual projections onto the Time-4 PCA model were calculated for the rest of times. In this way, samples with values below the confidence limit are considered similar to those of Time 4, that is, ready to be harvested.

**Figure 4:**
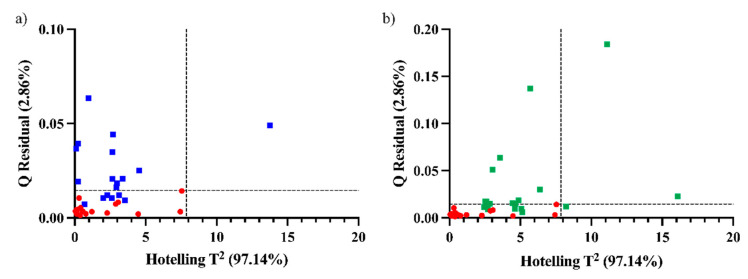
Q residual and Hotelling T^2^ for Time 4 (red circles) and the projection in the model of (**a**) Time 3 (blue squares) and (**b**) Time 5 (green squares).

As spatial position factors affect the composition of the grapes, each time had its own variability between the parts of the plant and bunch. It is possible that for times nearer to Time 4, some of the grapes, i.e., some positions, had the same spectral characteristics of Time 4. As expected, all samples from the first two times are above the confidence limit in one or both statistics, thus showing very different characteristics from the samples belonging to the time of harvest (Time 4). For times nearer to the harvest time, some of the samples lie below the confidence limit (Figure 4). Six and five samples lie below the limits for Time 3 and 5, respectively. It is worthwhile to mention that the samples that lie below the limits are not the same in Times 3 and 5 in terms of position in the plant and in the bunch. This would mean a maturity evolution of certain parts of the plant for Time 3 that are similar to Time 4 grapes and certain parts of the plant for Time 5 that are similar to Time 4. However, since there is no uniform behavior in the different positions, we cannot directly decide from the control charts which bunches to harvest already at Time 3 or which ones will be overripe at Time 5.

From all the presented results we can conclude that the proposed methodology could be used as a quality control chart to select the grapes that are ready to harvest. Thus, this methodology could assess which parts of the plant are ready to be harvested at Time 3 or even wait to be harvested at Time 5; however, it will require more samples in the future and that the study is performed in a specific vineyard to guarantee accurate results.

## 4. Conclusions

In this study, a fast and reliable grape ripening control methodology has been developed using a portable ATR-MIR instrument. This methodology has made it possible to predict basic oenological parameters (TSS and pH), which enables the technological maturity of the grapes to be determined. In addition, the different sources of variability in the evolution of grape ripening were also studied using ASCA and it has been objectively shown that the position of the grape, both in the vine and in the bunch, significantly affect ripeness. These results support the idea of splitting the harvest into different days to achieve the best possible quality for each grape. For that reason, we also proposed a control chart to determine which grapes have the optimal characteristics to be harvested at each moment. The results obtained, although preliminary, have shown that this chart could be a useful tool for viticulturists to make the most appropriate decisions about which parts of the vine should be harvested at any given time.

## Figures and Tables

**Table 1:** Results of the control of ABV (potential alcoholic strength by volume, expressed in alcoholic degrees), pH, TPC (total polyphenolic content, expressed in mg of gallic acid equivalent per 100 mL) and ORAC (oxygen radical absorbance capacity, expressed in μmol of Trollox equivalent per 100 mL). * Values not measured. Different letters indicate significant differences (*p*-value < 0.05).

Sampling Point	ABV	pH	TPC	ORAC
T1	9.6 ± 1.5 ^a^	3.15 ± 0.08 ^ab^	*	*
T2	11.0 ± 1.1 ^b^	3.10 ± 0.10 ^a^	10.3 ± 0.6 ^a^	437 ± 89 ^a^
T3	12.1 ± 0.4 ^c^	3.28 ± 0.08 ^b^	16.8 ± 1.4 ^c^	620 ± 125 ^b^
T4	12.4 ± 0.4 ^c^	3.33 ± 0.13 ^b^	16.2 ± 1.4 ^c^	614 ± 125 ^b^

**Table 2:** Prediction results of total soluble solids (TSS) and pH for different validation strategies. RMSEP values for TSS are expressed in Brix. (R^2^_Pred_ = determination coefficient of the regression line between predicted and measured values, RS = region selection, LV = number of latent variables of the model), KS = Kennard–Stone algorithm to select the validation set, onion = onion algorithm to select the validation set.

	TSS (°Brix)			TSS (°Brix)^RS^			pH		
	RMSEP	R^2^_Pred_	LV	RMSEP	R^2^_Pred_	LV	RMSEP	R^2^_Pred_	LV
CV random	0.3	0.982	3	0.3	0.981	2	0.07	0.683	9
KS (½ cal–½ val)	0.3	0.984	3	0.3	0.962	2	0.06	0.623	9
KS (⅔ cal–⅓ val)	0.3	0.984	3	0.3	0.979	2	0.06	0.608	9
Onion (½ cal–½ val)	0.3	0.985	3	0.4	0.980	2	0.07	0.687	9
Onion (⅔ cal–⅓ val)	0.3	0.986	3	0.4	0.971	2	0.07	0.591	9

**Table 3:** Main effects and their combinations for the ASCA model.

Term	% Effect	*p*-Value
Maturity (sampling date)	28.98	0.0001
Position in the plant	5.83	0.0001
Position in the bunch	2.21	0.0268
Instrumental replicate	0.11	0.9116
Maturity × Position in the plant	9.52	0.0001
Maturity × Position in the bunch	5.84	0.0001
Maturity × Instrumental replicate	0.10	1.0000
Position in the plant × Position in the bunch	2.14	0.0224
Position in the plant × Instrumental replicate	0.15	0.9969
Position in the bunch × Instrumental replicate	0.11	0.9987

**Table 4:** Results of the ASCA models for each individual harvest period. T1 to T5: number of the different sampling times. * = Significant effect of the factor (*p* < 0.05).

	% Effect				
Sampling Times	T1	T2	T3	T4	T5
Position in the plant	7.41	40.75 *	3.67	36.57 *	11.92 *
Position in the bunch	22.00 *	19.90 *	5.52	2.32	5.27
Instrumental replicate	0.23	0.14	0.57	0.28	0.38
Position in the plant × Position in the bunch	46.95 *	11.18 *	28.98 *	4.61	23.97 *
Position in the plant × Instrumental replicate	0.44	0.47	1.18	0.23	0.51
Position in the bunch × Instrumental replicate	0.47	0.61	0.95	0.52	0.54
Residual	22.50	26.95	59.14	55.48	57.42

**Table 5:** Main effects and their combinations for the ASCA model.

Term	% Effect	*p*-Value
Maturity (sampling date)	40.49	0.0001
Position in the plant	4.03	0.0300
Position in the bunch	3.10	0.0657
Maturity × Position in the plant	9.07	0.0563
Maturity × Position in the bunch	3.38	0.7729
Position in the plan × Position in the bunch	2.67	0.3663
Residuals	37.25	-

## Data Availability

The data presented in this study are available on request from the corresponding author.

## Supplementary Materials

The following supporting information can be downloaded at: https://www.mdpi.com/article/10.3390/foods12050962/s1, Table S1: Maturity, position in the plant and position in the bunch factors and their levels; the number of samples; TSS and pH values (mean ± standard deviation) of each level of each factor. Different letters mean significant differences between the levels of the factor. Figure S1: (a) PC2 vs PC1 score plot and (b) Q residual versus Hotelling T2 plot of the ATR-FTIR spectra for crushed grapes and (c) DRIFT for intact grape. Figure S2: Plots of the first factor of the position in the plan, position in the bunch, interaction maturity x position in the plant, interaction maturity x position in the bunch and interaction position in the plant x position in the bunch factors submodels. Figure S3: Q residual and Hotelling T2 for Time 4 (red circles) and the projection in the model of (a) Time 1 (purple squares) and (b) Time 2 (yellow squares).
