# Supplementary material for: Assessment of Variability Sources in Grape Ripening Parameters by Using FTIR and Multivariate Modelling
Source: Foods. 2023 Feb 24;12(5):962. doi: 10.3390/foods12050962 (PMC10001218; doi:10.3390/foods12050962)
Supplement: Supplementary file 1 [file foods-12-00962-s001.zip › foods-2190314- supplementary_checked.pdf]

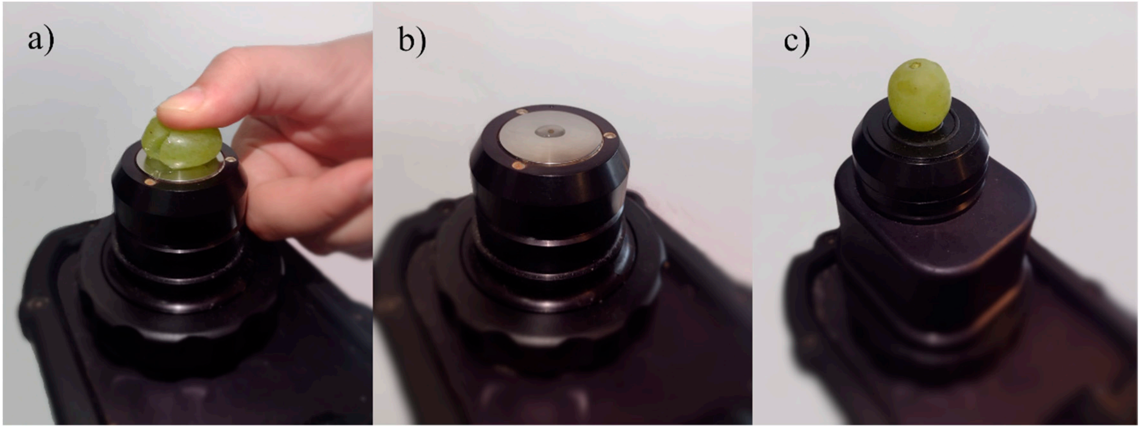

Figure S1. Illustrative photos taken during the analysis of grapes using the different configurations: a) ATR-FTIR for intact grape; b) ATR-FTIR for crushed grape; and c) DRIFT for intact grape.

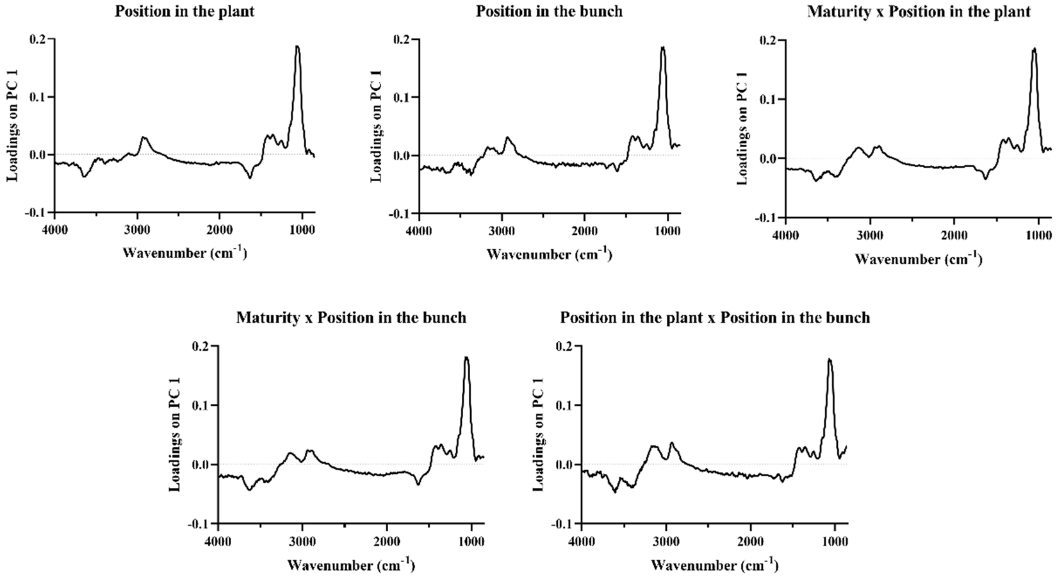

Figure S2. Plots of the first factor of the position in the plant, position in the bunch, interaction maturity x position in the plant, interaction maturity x position in the bunch and interaction position in the plant x position in the bunch factors submodels.

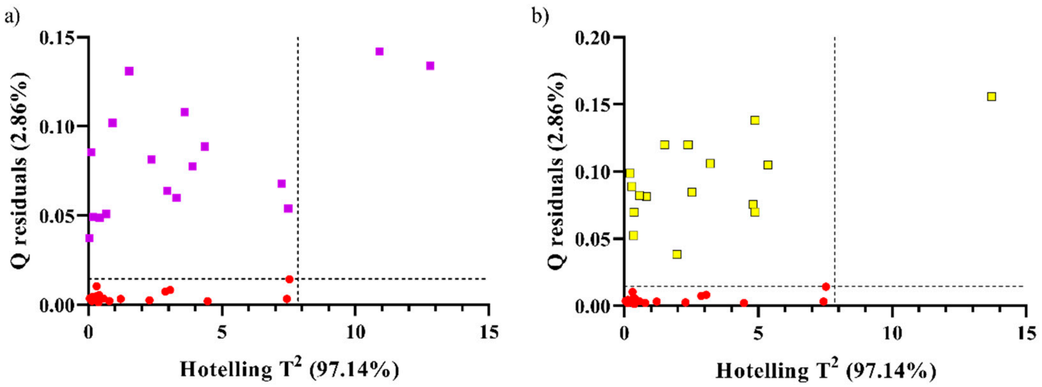

Figure S3. Q residual and Hotelling T^2 for Time 4 (red circles) and the projection in the model of (a) Time 1 (purple squares) and (b) Time 2 (yellow squares).
