# Supplementary material for: Assessment of Variability Sources in Grape Ripening Parameters by Using FTIR and Multivariate Modelling
Source: Foods. 2023 Feb 24;12(5):962. doi: 10.3390/foods12050962 (PMC10001218; doi:10.3390/foods12050962)
Supplement: Supplementary file 1 [file foods-12-00962-s001.zip › Supplementary Table.pdf]

Table S1. Maturity, position in the plant and position in the bunch factors and their levels; the number of samples; TSS and pH values (mean ± standard deviation) of each level of each factor. Different letters mean significant differences between the levels of the factor.

**Factor**	**Level**	**# Samples**	**TSS**	**pH**
Maturity	1	18	16.4 ± 2.6 a	3.15 ± 0.08 ab
	2	18	18.8 ± 2.1 b	3.10 ± 0.11 a
	3	18	20.7 ± 1.3 c	3.28 ± 0.11 cd
	4	18	20.6 ± 1.8 bc	3.36 ± 0.13 d
	5	18	21.2 ± 1.9 c	3.25 ± 0.09 bc
Position in the plant	Top	30	18.6 ± 2.9 a	3.19 ± 0.15 a
	Middle	30	19.5 ± 2.8 a	3.24 ± 0.14 a
	Bottom	30	20.2 ± 2.0 a	3.23 ± 0.12 a
Position in the bunch	Top	30	19.6 ± 2.6 a	3.25 ± 0.16 a
	Middle	30	19.4 ± 2.6 a	3.24 ± 0.13 a
	Bottom	30	19.3 ± 2.7 a	3.17 ± 0.12 a
